# The eye’s coral reef: toward a planetary-health agenda for ocular-microbiome stewardship

**DOI:** 10.3389/fmicb.2026.1816460

**Published:** 2026-04-08

**Authors:** Lorenzo Drago, Luigi Regenburgh De La Motte

**Affiliations:** 1 Laboratory of Clinical Medicine with Specialized Areas, IRCCS MultiMedica, Milan, Italy; 2 Clinical Microbiology and Microbiome Laboratory, Department of Biomedical Sciences for Health, University of Milan, Milan, Italy

**Keywords:** low-biomass ecosystems, microbial ecology, microbial resilience, ocular microbiome, planetary health

## Abstract

Coral reefs and the human ocular surface represent ecologically distinct yet structurally comparable microbial ecosystems in which resilience depends on finely regulated host–microbe interactions. In coral reef science, microbial shifts precede visible bleaching and ecosystem collapse, enabling the development of predictive stress indices such as Degree Heating Weeks (DHW). Comparable principles are emerging in host-associated, low-biomass microbiomes, where subtle perturbations may trigger disproportionate functional consequences. Here, we propose a systems-level conceptual framework linking coral reef holobionts and the ocular surface as sentinel ecosystems governed by cumulative stress, threshold dynamics, and microbial instability. We introduce two heuristic constructs—the Cumulative Desiccating Load (CDL) and the Ocular Dysbiosis Sentinel Index (ODSI)—to frame dysbiosis as a trajectory of resilience loss driven by cumulative perturbations. Aging-related conditions such as age-related macular degeneration are discussed as examples of microbial and metabolic senescence within the human holobiont, conceptually paralleling coral reef decline under chronic sublethal stress. By integrating environmental and host-associated microbiome research within a planetary-health perspective, this article advances a resilience-oriented systems framework applicable across biological scales.

## 1 Introduction

Microbial communities are central determinants of ecosystem stability, whether in complex environmental holobionts or host-associated biological niches. Across systems, resilience depends on dynamic host–microbe equilibrium, and collapse often follows cumulative rather than acute perturbations. Coral reefs provide one of the most extensively studied examples of stress-sensitive ecosystems in which microbial shifts precede visible structural failure. Decades of research have demonstrated that thermal stress, acidification, and eutrophication destabilize coral-associated microbial communities prior to bleaching events, prompting the development of integrative stress metrics such as Degree Heating Weeks (DHW) (NOAA Coral Reef Watch and ICRI, 2025; Hughes et al., 2017). Comparable dynamics are increasingly recognised in host-associated microbiomes, particularly in low-biomass systems where subtle microbial perturbations can precipitate disproportionate functional consequences. In this Perspective, we examine coral reef holobionts and the ocular surface microbiome as representative stress-sensitive systems to explore shared principles of cumulative stress, threshold dynamics, and resilience loss within a systems microbiology framework.

## 2 Sentinel microbiomes across biological scales

Coral reefs are among the most visible fragile ecosystems: kaleidoscopic when balanced, ghostly when bleaching foreshadows collapse. Marine science has developed integrative approaches to quantify cumulative stress. DHW capture thermal load over time, pulse-amplitude fluorometry tracks photosynthetic decline, and microbial shifts—often characterised by the loss of *Endozoicomonas* and the expansion of *Vibrio* spp. anticipate disease outbreaks (Bourne et al., 2009; Voolstra et al., 2024). Composite indices such as the Coral Health Index (CHI) integrate physical, biological, and microbial parameters into prognostic tools of ecological frailty. A parallel lexicon is emerging in ophthalmology. The Tear Film and Ocular Surface Society Dry Eye Workshop II (TFOS DEWS II) defined dry eye disease as a failure of tear-film homeostasis driven by instability, hyperosmolarity, inflammation, and neurosensory abnormalities—an ecological definition at its core (Craig et al., 2017). Tear osmolarity, matrix metalloproteinase-9 (MMP-9), lactoferrin, and microbial diversity metrics serve as indicators of surface instability (Bron et al., 2017). Commensals such as *Corynebacterium mastitidis*, *Staphylococcus epidermidis*, and *Streptococcus mitis* contribute to mucosal resilience (St Leger et al., 2017). Despite differences in scale and biodiversity, both reef and ocular surface function as sentinel ecosystems in which early microbial and inflammatory perturbations signal broader environmental or biological imbalance. Framed within systems microbiology, both represent host–microbe assemblages governed by feedback regulation, threshold responses, and nonlinear transitions under cumulative stress.

## 3 Cumulative stress and threshold dynamics

Rather than interpreting dysbiosis as isolated microbial fluctuation, cumulative perturbations may drive systems transitions toward alternative stable states. In coral reefs, DHW thresholds translate thermal stress into predictive insight: values exceeding 4 °C-weeks signal stress onset, while values above 8 °C-weeks predict widespread bleaching and mortality. Analogously, we propose the heuristic concept of Cumulative Desiccating Load (CDL) to integrate upstream pressures acting on the ocular surface—including screen exposure, contact lens wear, ambient dryness, pollution, systemic medications, and preserved eye drops. Complementarily, the Ocular Dysbiosis Sentinel Index (ODSI) integrates downstream indicators of microbial stability, inflammatory burden, and host-protective factors. CDL and ODSI are not diagnostic tools but conceptual frameworks that model trajectories of resilience loss and recovery. By translating cumulative stress into system-level indicators, they parallel reef-style dashboards that convert environmental perturbation into ecological foresight (Kahook et al., 2024).

## 4 Aging, microbial senescence, and systems fragility

Aging provides a unifying lens through which cross-scale microbial resilience becomes evident. In the eye, age-related macular degeneration (AMD) exemplifies a condition of imbalance rather than infection, characterised by oxidative stress, mitochondrial dysfunction, and chronic inflammation. Evidence suggests extension of this instability along the gut–eye axis, where systemic dysbiosis alters metabolic and immunological tone (Xiao et al., 2023; Grant et al., 2022). Reductions in butyrate-producing taxa and increases in pro-inflammatory genera accompany altered short-chain fatty acid profiles, suggesting participation of microbial metabolites in retinal inflammaging loops. Coral reefs undergo comparable ecological aging. Prior to bleaching, microbial senescence manifests as shifts from mutualistic consortia (*Endozoicomonas, Ruegeria*) toward opportunistic assemblages (*Vibrio, Alteromonas, Tenacibaculum*). Chronic sublethal warming and eutrophication reduce holobiont stability and lower collapse thresholds. Across both systems, loss of microbial diversity, impaired antioxidant defenses, and inflammatory amplification represent emergent properties of system fragility under cumulative stress.

## 5 Discussion

This Perspective proposes that host-associated low-biomass microbiomes and complex environmental holobionts share systems-level properties, including cumulative stress integration, feedback-mediated instability, threshold transitions, and resilience loss. By framing the ocular surface as a model sentinel ecosystem, we extend principles derived from coral reef ecology to host-associated microbiomes without implying equivalence in biodiversity or scale. The value of this comparison lies not in analogy alone, but in highlighting shared mechanisms of microbial resilience across biological systems. Future research should focus on developing quantitative systems models capable of integrating microbial network dynamics, host responses, and environmental perturbations across scales. Such integrative approaches align with planetary health frameworks in which microbiome monitoring serves as an early-warning strategy for ecosystem fragility. Recognising dysbiosis as a systems transition rather than a static state may shift emphasis from reactive repair toward resilience preservation. In both corals and humans, stewardship becomes a method grounded in protecting host–microbe symbiosis before collapse becomes irreversible.

## Data Availability

The original contributions presented in the study are included in the article/supplementary material, further inquiries can be directed to the corresponding authors.
